# Role of Solvents in Iron Nanoparticle Synthesis: Analyzing Water and 1‑Methyl-2-Pyrrolidone with Green Tea Extract as a Reducing Agent

**DOI:** 10.1021/acsomega.5c03914

**Published:** 2025-07-10

**Authors:** Nikhil Kumar Daimari, Ashish Gogoi, Rajib Biswas, Nirmal Mazumder

**Affiliations:** † Applied Optics and Photonics Lab, Department of Physics, 28688 Tezpur University, Tezpur, Assam 784028 India; ‡ Department of Biophysics, Manipal School of Life Sciences, 76793 Manipal Academy of Higher Education, Manipal, Karnataka 576104, India

## Abstract

The choice of solvent in the synthesis of nanoparticles plays a pivotal role in influencing the nucleation and growth kinetics of nanoparticles. Deionized water (DI) due to its cost-effectiveness, low toxicity, and ability to dissolve precursor salts effectively makes an ideal solvent medium, while aprotic organic solvents such as *N*-methyl-2-pyrrolidone (NMP) with high dipole moment also demonstrate their efficacy as dual solvent-reducing agents. Herein, this study aims to explore the effect of different solvent media on the biosynthesis of iron nanoparticles (FeNPs) and their impact on the optical, structural, and morphological properties. Green tea extract acts as a reducing agent aiding in stable nanoparticle formation by surface capping with active phytochemical functional groups. The synthesized FeNPs were characterized using ultraviolet–visible (UV–vis) spectroscopy, X-ray diffraction (XRD), field emission scanning electron microscopy (FESEM), energy-dispersive X-ray (EDX), zeta sizer, and Fourier transform infrared (FTIR) spectroscopy. The appearance of absorption peaks affirmed ligand-to-metal charge transfer and double exciton transitions undergoing in the optical structure of the nanoparticles. XRD analysis confirmed the formation of a mixed-phase hematite (α-Fe_2_O_3_) and maghemite (γ-Fe_2_O_3_) nanostructure with rhombohedral and cubic lattices. Morphological studies by FESEM specify high-yield synthesis of FeNPs with mean particle size of 52.20 ± 14.65 and 51.77 ± 13.82 nm for DI and NMP, respectively. The oxidation of NMP solvent molecules also functioned as a coreducing agent for the reduction of metal Fe species allowing the growth of FeNPs at ambient room temperature. The effectiveness of NMP in FeNPs synthesis highlights its potential as a practical route for producing iron-based nanomaterials while revealing key aspects of solvent–nanoparticle interactions.

## Graphical abstract

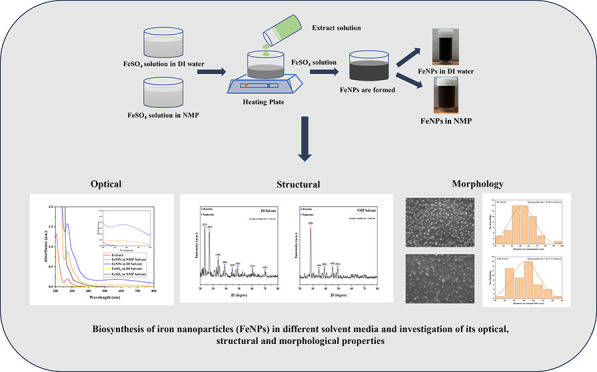

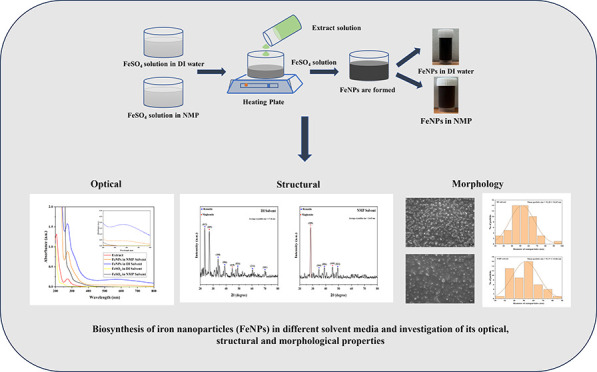

## 1 Introduction

Iron-based nanomaterials have garnered widespread attention due to their exceptional properties such as magnetic behavior, catalytic potential, large specific surface area, and heightened reactivity.^1^ Their applications span from efficient adsorption of heavy metals to the photocatalytic degradation of harmful dyes highlighting their versatility.^2^ Diverse methods of synthesis are employed for the preparation of iron-based nanomaterials. In recent years, green synthesis has garnered significant interest owing to its environmentally friendly approach and the potential for producing stable nanomaterials without the use of toxic chemicals. These methods are safer, environmentally benign, and cost-effective and do not involve toxic reagents. For instance, the use of plant extracts, which are rich in phytochemicals such as polyphenols, flavonoids, terpenoids, and phenolic acids, is widely popular for reducing metal ions and stabilizing nanoparticles. Similarly, some biological components such as enzymes act as reducing and capping agents, facilitating large-scale nanoparticle production.^3,4^ In addition, the process of green synthesis minimizes waste and pollution with no unwanted byproducts while also eliminating the need for hazardous solvents, making it ideal for large-scale, eco-friendly production. Another advantage of green synthesis of metal oxide NPs is that it requires only fewer purification steps without any aggressive procedures such as vacuum conditions, high pressure, and high energy.^5^ While considerable research has focused on the synthesis, physicochemical properties, and applications of metal and metal oxide nanoparticles, their environmental implications still remain a critical area of study.

During nanoparticle synthesis, the selection of an appropriate solvent is a critical factor, as it significantly influences particle formation, growth, and stability. Several key solvent properties, such as dipole moment, dielectric constant, acceptor and donor capabilities, solubility, and cohesive pressure, must be considered to ensure efficient dissolution of precursor salts and intermediates, thereby facilitating uniform nucleation and controlled growth processes.^6^ Water is widely recognized as an ideal solvent in nanoparticle synthesis due to its cost-effectiveness, broad availability, and nontoxic nature.^7^ Owing to its capacity to dissolve ionic precursors and minimization of hazards associated with organic solvents, it is a highly suitable solvent for sustainable synthesis.

In addition to traditional aqueous synthesis methods, the use of organic solvents such as 1-methyl-2-pyrrolidone (NMP) has emerged as a viable alternative for synthesizing iron nanoparticles (FeNPs). NMP being a polar aprotic solvent possesses a strong polarity (μ = 4.09 D)^8^ which can effectively dissolve various precursors such as polar and ionic species and stabilize nanoparticles during synthesis. The presence of nonpolar carbon atoms and a large planar nonpolar region in NMP facilitates hydrophobic interactions with nonpolar molecules, leading to complex formation.^9^ This distinctive structural characteristic underpins its unique physicochemical properties^10^ and supports its widespread use as a solvent, cosolvent, and complexing agent across various applications, including the pharmaceutical and electronics industries.^9,11,12^ Additionally, several studies have demonstrated that NMP exhibits significant potential as an effective scavenger of oxidizing agents.^13^ Although NMP offers several advantages, its potential as a medium for the synthesis of metal nanoparticles has not been systematically explored. In a study, Amgoth and colleagues synthesized spherical gold nanoparticles (AuNPs) in an NMP solution by employing sodium citrate as a strong reducing agent under heating conditions. Their findings indicated that using NMP as a solvent enhances the polarity of the medium, facilitating the formation of smaller AuNPs.^14^ In another separate study, Esfahani et al. synthesized gold nanostructures (AuNS) using NMP as both reducing agent and solvent media in the presence of poly­(vinylpyrrolidone) (PVP). Their results concluded that stable AuNS were formed in the organic medium with high yield demonstrating the efficacy of NMP acting as a dual solvent-reducing agent without any pretreatment of NMP.^15^ Furthermore, in our previous work, we have also reported the synthesis of silver nanoparticles (AgNPs) in different solvent media. It was found that both green and chemically synthesized AgNPs in NMP medium showed prominent surface plasmon resonance peaks indicating the formation of AgNPs with FCC crystal structure.^16^

Understanding the interactions between the solvent and the synthesized nanoparticles is crucial for optimizing production processes and enhancing their performance in diverse applications.^7,10,17^ Although there is abundant literature detailing the synthesis procedure, very few articles address the impact of solvent on the biogenic synthesis of FeNPs. In an attempt to shed light on this research gap, this study reports the biosynthesis of FeNPs in different solvent media, namely, deionized water (DI) and NMP. The study aims to focus on the effect of solvent media which can influence the optical, structural, and morphological properties of FeNPs.

## 2 Materials and Methods

### 2.1 Materials

Green tea of Lipton brand was purchased from a local market. Analytical grade iron­(II) sulfate heptahydrate (FeSO_4_·7H_2_O) (≥98.5%) and 1-methyl-2-pyrrolidone (NMP) (≥99.5%) were purchased from Merck Life Science Private Limited, India. Reagent grade sodium hydroxide (NaOH) was obtained from Loba Chemie Private Limited, India. Whatman filter paper no. 1, syringe filter of 0.22 μm, and deionized water (DI) were used. For cleaning purposes, freshly prepared aqua regia and distilled water (DW) were used.

### 2.2 Instruments

After synthesis, initial characterization of the material was done using an ultraviolet–visible (UV–vis) spectrophotometer (Thermo Scientific GENESYS 180) at room temperature. The structural analysis was done using an X-ray powder diffractometer (model: D8 FOCUS, make: Bruker AXS, GERMANY) in a scan range of 20–80° with a scanning interval of 2θ = 0.10°. The morphology of the samples was observed by a field emission scanning electron microscope (JEOL, JSM7200F) with an accelerating voltage of 15 kV. Elemental analysis of the sample was performed via EDX (JEOL, JSM 6390LV) while the hydrodynamic size distribution was observed using a Zeta Sizer (Malvern Analytical, Nano ZS90). The FTIR spectra were recorded on a Fourier transform infrared spectrometer (PerkinElmer, SPECTRUM 100), within the wavenumber range of 4000–400 cm^–1^ to investigate the possible changes in the functional groups of FeNPs. The samples were finely ground, blended with KBr, and compressed into a pellet form for analysis. A weighing machine (METTLER TOLEDO, ME204), a centrifuge machine (Eppendorf 5430R), an oven (Ecogian series, EQUITRON), and a magnetic stirrer (SPINOT-TARSONS) were also used in this work.

### 2.3 Preparation of Green Tea Extract

First, 80 mL of DI was heated for a period of 15 min in a 100 mL beaker to 100 °C. One gram of green tea powder of brand Lipton was ground into fine particles and added to the heated DI and stirred using a glass rod. The mixture was then kept for 15 min to allow the release of tea polyphenols and flavonoids into DI. The prepared extract solution was then double filtered using Whatman filter paper no. 1. The light-yellow supernatant was collected and stored at 4 °C for further use.

### 2.4 Biosynthesis of Iron Nanoparticles (FeNPs) in Different Solvents

The synthesis of iron nanoparticles was done using green tea extract as a reducing agent in two different solvents, namely, DI and NMP. Stock solutions of 0.1 M iron sulfate heptahydrate (FeSO_4_·7H_2_O) were prepared by adding 2.78 g of FeSO_4_·7H_2_O to 100 mL of DI and NMP solution. The solution was stirred for 15 min until complete dilution. Subsequently, 20 mL of extract solution was taken in a different beaker, and to it 10 mL of 0.1 M FeSO_4_·7H_2_O solution was added in a 2:1 ratio under stirring at room temperature while maintaining a pH of 7 by the addition of NaOH. The solution was continuously stirred for 30 min. A change in color from transparent yellow to black confirmed the formation of biosynthesized iron nanoparticles in DI solvent. Similarly, the same procedure was followed for the synthesis of FeNPs in NMP solvent. The nanoparticle solution was centrifuged at 13,000 rpm for 30 min and eventually kept for drying in an oven at 60 °C for 12 h for further analysis (Figure 1).

**Figure 1:**
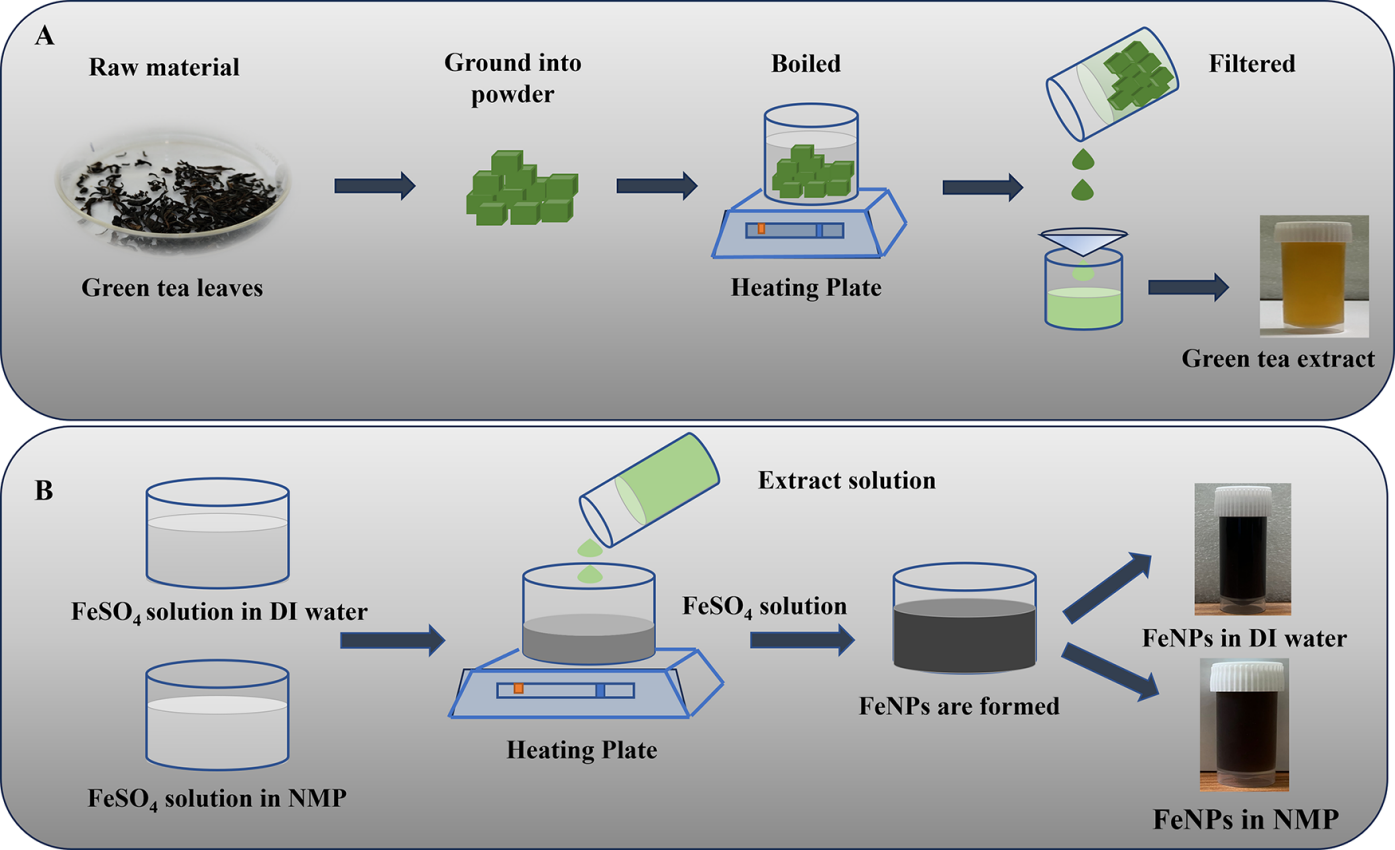
Schematic representation of (A) preparation of green tea extract and (B) biosynthesis process of FeNPs in different solvents.

## 3 Results and Discussion

Initially, UV–visible spectroscopy was employed to analyze the absorption spectra of biosynthesized FeNPs in both DI and NMP solvents. The absorption spectrum (Figure 2) revealed characteristic peaks at 272 nm in DI solvent and 274 nm in NMP solvent, respectively, indicating a reaction between Fe^2+^ and green tea extract where the polyphenols present played a crucial role in the reduction of Fe^2+^ ions to nanoparticles which resulted in a rapid color change from yellow to black. Polyphenols in green tea extract reduce Fe^2+^ ions to form Fe^0^ or Fe oxide nanoparticles and simultaneously cap the nanoparticle surface, stabilizing them against aggregation. This capping is facilitated by the hydroxyl (−OH) groups and aromatic rings in polyphenols, which form strong bonds with the nanoparticle surface via chelation and hydrogen bonding. The slight shift in peaks from 272 nm (DI) to 274 nm (NMP) can be attributed to the minor differences in particle size of the nanoparticles.^18^ The absorption peaks observed in the UV regions are due to ligand-to-metal charge transfer which occur from the nonbonding ligand molecular orbitals (O 2p) to the antibonding partially filled metal d-orbitals (Fe 3d).^19^ A broad absorption band was also observed in the wavelength range of 500–700 nm as shown in the inset of Figure 2 confirming the formation of FeNPs. This broad absorption in the visible region is characteristic of double exciton processes involving strongly coupled Fe^3+^ cations.^19^ The findings in this study align well with previously reported data.^45,46^ Additionally, it was noted that FeNPs biosynthesized in DI showed a strong absorption band compared with those synthesized in NMP indicating higher reactivity of green tea extract with Fe^2+^ in DI as a solvent. Furthermore, polar functional groups and hydrogen bonding abilities present in the solvents such as NMP and DI, respectively, also enable the dissociation of ionic compounds present, increasing their solubility for consistent nucleation. Besides, the polar solvent molecules possess the ability to donate electrons through their amide functional groups contributing to the stabilization of the resulting charged nanoparticles, thereby facilitating NMP to perform as a coreducing agent, suggesting the formation of FeNPs in NMP solvent.^16,20^ Protic solvents such as DI water exhibit strong hydrogen bonding capabilities and a high dielectric constant (∼80), which promote the ionization of surface groups on nanoparticles (e.g., hydroxyl or carboxyl groups). This leads to increased surface charge, enhancing electrostatic repulsion between particles and reducing aggregation. This results in sharper peaks and reduced base broadening due to a more uniform particle size distribution. In contrast, aprotic solvents such as NMP lack hydrogen bonding capabilities and have a lower dielectric constant (∼32), which may result in reduced ionization of surface groups and weaker electrostatic stabilization. This often causes peak red shifting (indicative of larger or aggregated particles) and base broadening due to a broader size distribution or structural inhomogeneities.^21–23^ It is known that under specific conditions, NMP can undergo oxidation to yield 5-hydroxy-*N*-methyl-2-pyrrolidone, which may further oxidize into *N*-methyl succinimide and 2-hydroxy-*N*-methyl succinimide. During this oxidation pathway, initially unstable peroxide intermediates are generated, which subsequently convert NMP into 5-hydroxy-*N*-methyl-2-pyrrolidone. This secondary alcohol can function as a reducing agent in the presence of metal ions, promoting nanoparticle synthesis while itself being further oxidized to *N*-methyl succinimide.^15,24^

**Figure 2:**
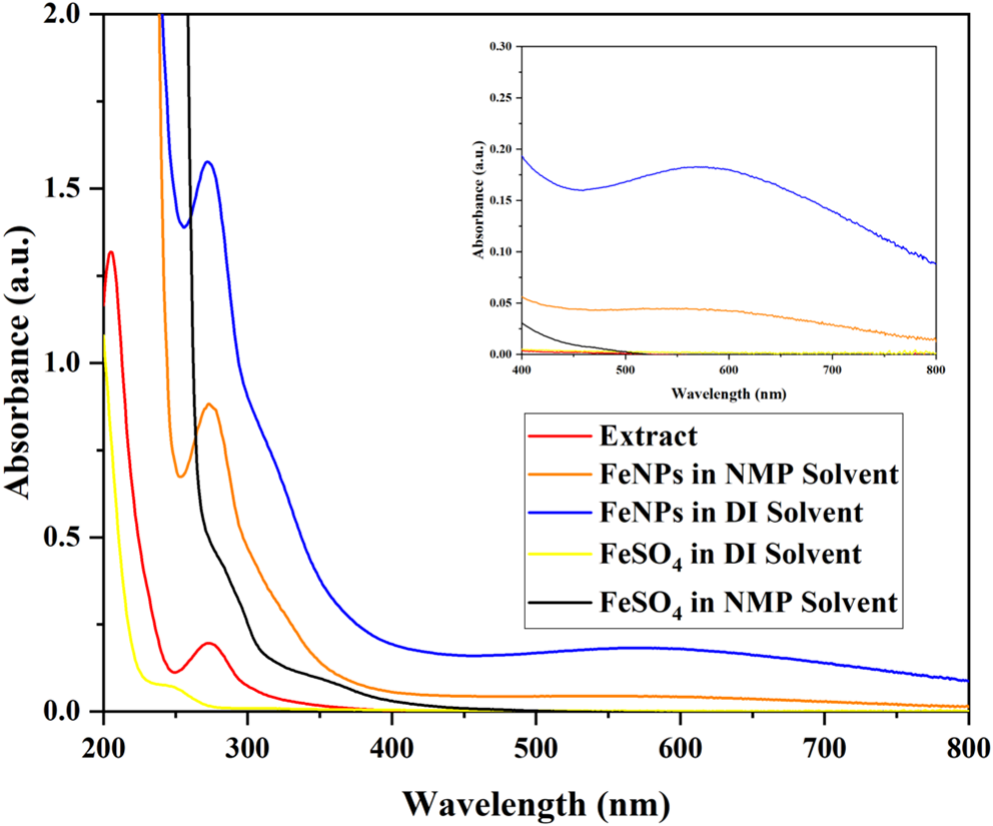
UV–visible spectrum of green tea extract and FeNPs in solvent DI and NMP. The inset displays the broad absorption band.

The stability of FeNPs shown in Table 1 was evaluated in both DI and NMP solvents at room temperature over a period of 10 days. The maximum wavelength value was found to remain the same under the same storage conditions (4 °C). However, the absorbance value showed a decrease in the value over the observed time period.

**Table 1:** Indicating the Stability of FeNPs in DI and NMP Solvents

FeNPs in DI solvent		
days	maximum wavelength (nm)	absorbance (a.u.)
1	272	1.57
5	272	1.45
10	272	1.38

FeNPs in NMP solvent		
days	maximum wavelength (nm)	absorbance (a.u.)
1	273	0.87
5	273	0.81
10	273	0.77

The optical band gap calculations were performed from the obtained UV–visible data using the Tauc relation given by the following equation:

1 $${\left(\alpha h\nu \right)}^{n}=C\left(h\nu -E_{g}\right)$$

In eq 1, α is the absorption coefficient, *C* is a constant, *hν* is the energy, and *n* is a constant depending on the nature of the electron transition.^25^ Reports suggest that iron oxide nanoparticles show evidence of both direct and indirect band gap material.^26,27^ Figures 3 and[Fig fig4] show the Tauc plot (α*hν*)*^n^* versus *hν* of FeNPs where *n* = 2 or 1/2 for direct allowed and indirect allowed transitions, respectively. The band gap energy was determined by extrapolating the linear fit region to the *x*-axis intercept in the case of a direct allowed transition. However, in the case of an indirect allowed transition, additionally a baseline correction method is followed by tracing a tangent to the curve just before the linear absorption curve.^28,29^ The intersection point of the two linear fitting lines determines the band gap for the indirect plot. The obtained optical band gap energies of FeNPs in DI and NMP solvents are listed in Table 2. The results showed significantly higher values than the typical band gap reported for bulk, which is around 2.2 eV. This increase in band gap is attributed to the quantum confinement effect. At the nanoscale size, the charge carriers are confined in smaller dimensions restricting the spatial movement of the charge carriers, leading to discrete energy levels with larger energy spacing between the levels. The increase in band gap can be quantified by the following equation:

2 $$E_{g,\mathrm{nano}}=E_{g,\mathrm{bulk}}+\frac{\pi^{2}\hbar^{2}}{{2R}^{2}}\left(\frac{1}{m_{e}^{*}}+\frac{1}{m_{h}^{*}}\right)-0.248E_{\mathrm{Ry}}^{*}$$

where in eq 2 *E*_g,nano_ is the optical band gap of the nanomaterial, *E*_g,bulk_ is the optical band gap of the bulk material, *E*_Ry_^*^ is the bulk exciton binding energy, *h* is the Planck’s constant, *R* is the radius of the nanomaterial, and *m*_e_^*^ and *m*_h_^*^ are the effective mass of electrons and holes, respectively. The second term represents the kinetic energy which rises inversely with *R* ^2^ significantly widening the band gap, thus revealing the crucial role of solvent in determining the electronic properties of synthesized FeNPs.^30,31^

**Figure 3:**
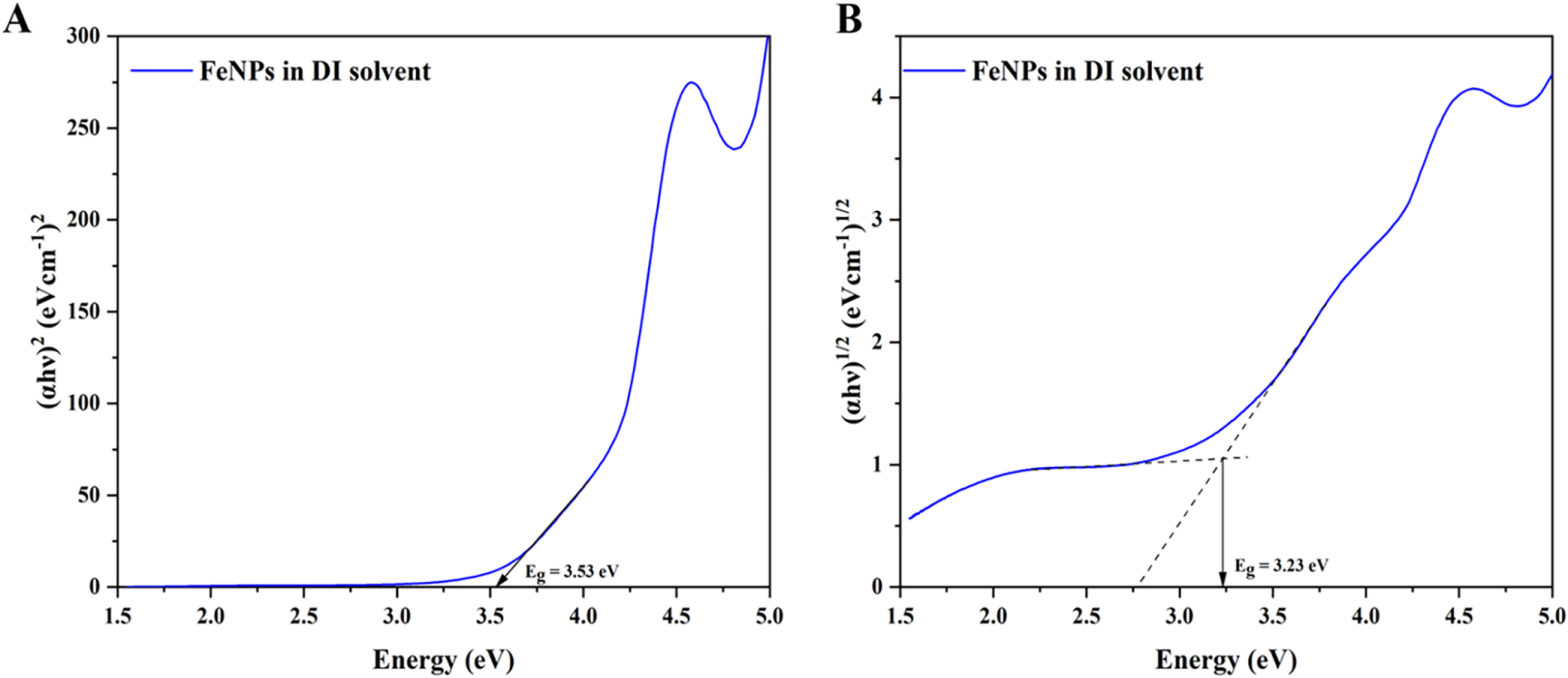
Tauc plots for (A) direct transition and (B) indirect transition in biosynthesized FeNPs in DI solvent.

**Figure 4:**
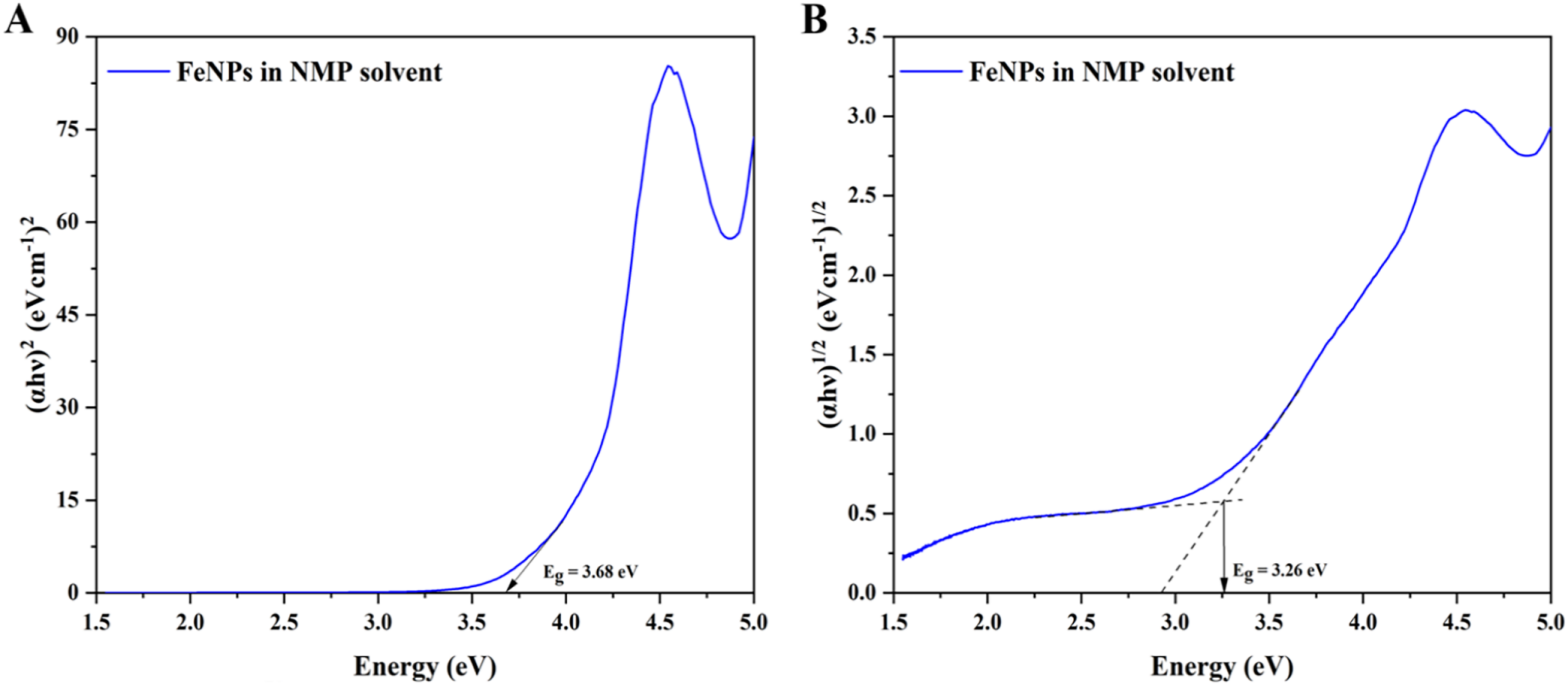
Tauc plots for (A) direct transition and (B) indirect transition in biosynthesized FeNPs in NMP solvent.

**Table 2:** Optical Band Gap Energy of FeNPs in DI and NMP Solvents

solvent	direct (eV)	indirect (eV)
DI	3.53	3.23
NMP	3.68	3.26

The XRD spectrum presented in Figure 5 confirms the crystallinity and phase composition of the synthesized FeNPs. A detailed analysis of the spectrum reveals that the material exists as a mixed phase of iron oxide nanoparticles, with phase identification performed using the standard JCPDS database. In Figure 5A, the diffraction peaks observed at 23.56° (012), 33.74° (104), 38.93° (006), 48.87° (024), 60.49° (214), and 70.07° (101) correspond to the rhombohedral phase of hematite (α-Fe_2_O_3_), as indexed in JCPDS card no. 01–079–0007.^32^ Additionally, two distinct peaks at 26.45° (205) and 45.02° (410) were identified, corresponding to the cubic phase of maghemite (γ-Fe_2_O_3_), referenced in JCPDS card nos. 01–039–1346 and 25–1402, respectively.^33,34^ However, in Figure 5B, it can be seen that diffraction peaks observed in NMP were less than those in DI solvent with the disappearance of the intense peak (012) confirming a lower degree of crystallinity in NMP solvent. These diffraction peaks at positions 28.49, 34.87, 39.04, 45.30, and 49.38° correspond to γ-Fe_2_O_3_ (205), α-Fe_2_O_3_ (104), α-Fe_2_O_3_ (006), γ-Fe_2_O_3_ (410), and α-Fe_2_O_3_ (024), respectively.^33,36^ It is evident that maghemite appears as an intermediate phase during the growth of hematite nanoparticles.^37^ The sharp peaks present indicated a crystalline nature.

**Figure 5:**
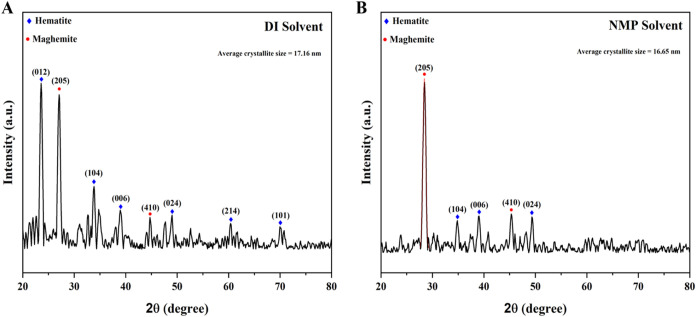
XRD spectra of FeNPs in (A) DI and (B) NMP solvents.

The crystallite size was calculated using the Debye–Scherrer equation.

3 $$D=\frac{k\lambda }{\beta \cos\theta }$$

In eq 3, λ = 1.5418 Å is the wavelength of Cu–K_α_ radiation, *k* = 0.9 is the shape factor, β is full width at half-maximum (FWHM) in radians, *D* is the diameter of the crystallite, and θ is Bragg’s angle.^38^ The average crystallite sizes of the prepared FeNPs in DI and NMP solvents were calculated to be 17.16 and 16.65 nm, respectively.

The surface morphologies of the biosynthesized FeNPs in various solvents were studied using field emission scanning electron microscopy (FESEM) as shown in Figure 6. It is evident from the images in Figure 6A that the nanoparticles were spherical and largely found to be aggregated and flocculated. Since no stabilizing agents were used during the synthesis process, the organic capping from the green tea polyphenols was insufficient to provide enough steric stabilization to overcome van der Waals attraction or magnetic forces leading to aggregation of the nanoparticles.^39,40^ In the presence of NMP solvent as shown in Figure 6B, FeNPs also showed aggregation as NMP has effectively less potential for surface ionization leading to higher surface energy, thus weakening electrostatic repulsion.^41,42^ Nonetheless, the flocculation is seen to be comparatively less in the NMP solvent. Additionally, Figure 6C,D displays the particle size distribution histogram plots from recorded FESEM data. The measured mean particle size for biosynthesized FeNPs was found to be 52.20 ± 14.65 and 51.77 ± 13.82 nm in DI and NMP solvents, respectively. It is known that the growth of nanoparticles occurs by the reduction of ions to zerovalent atoms, followed by the formation of seeds by primary nucleation and subsequently the formation of nanoparticles through secondary nucleation. This growth process is highly sensitive to temperature during the synthesis procedure, where elevated temperature within a controlled threshold tends to favor nucleation over particle growth.^43^ In this study, synthesis was carried out at ambient room temperature facilitating slower nucleation kinetics allowing for the development of larger nanoparticle seeds.^44^ It is evident that both DI and NMP solvents were favorable for nanoparticle formation.

**Figure 6:**
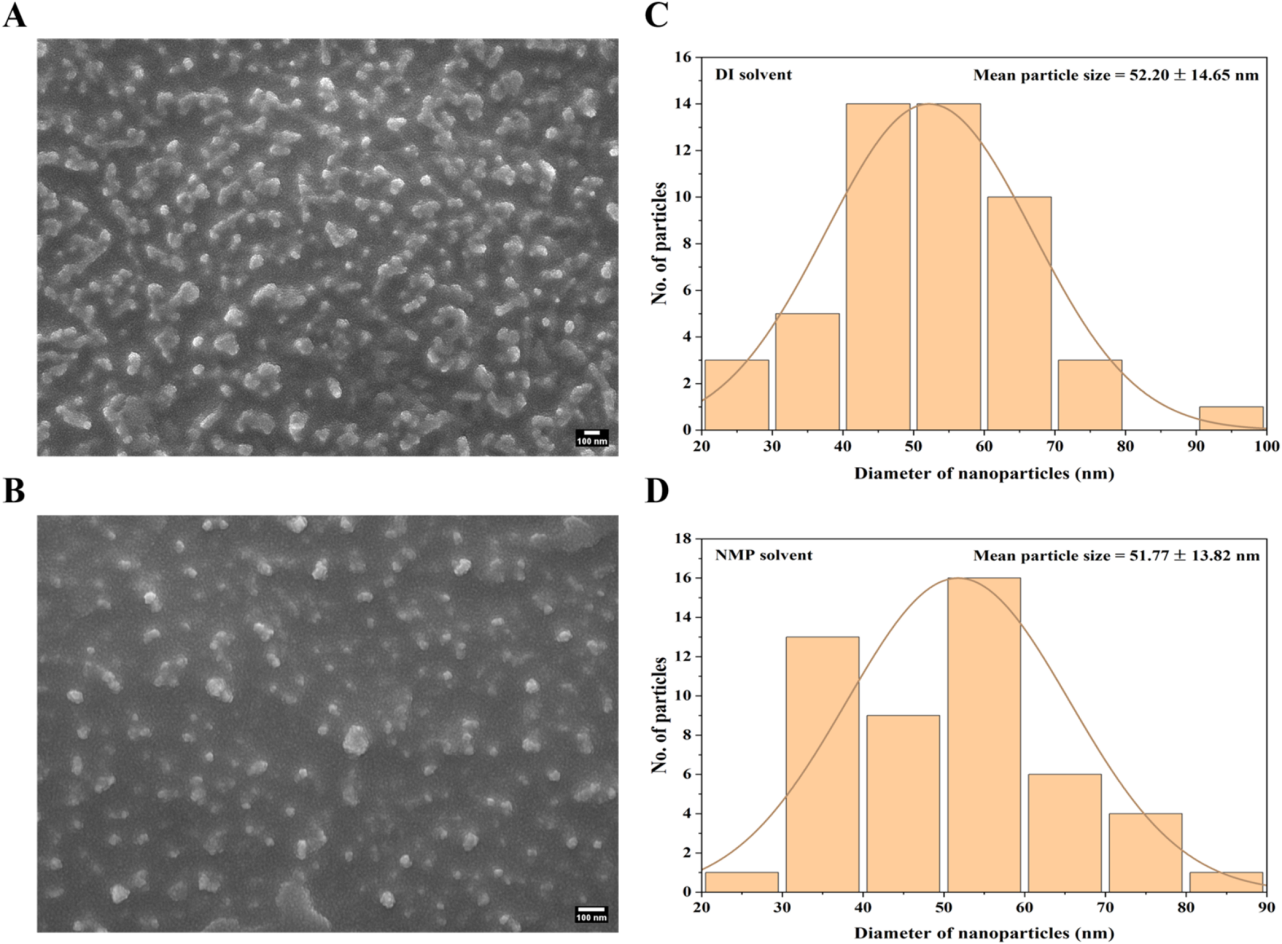
FESEM images of biosynthesized FeNPs in (A) DI solvent, (B) NMP solvent, and (C, D) their corresponding particle size distribution profile. Values are presented as mean ± standard deviation.

The elemental composition of FeNPs was determined by EDX experiments, as shown in Figure 7. The EDX spectra contain distinctive peaks of C and S in addition to Fe and O. The C and O peak signals are mainly because of the polyphenol groups and other biomolecules present in the green tea extracts.^45^ The S signals have originated from the use of iron sulfate heptahydrate (FeSO_4_.7H_2_O) precursor where residual sulfur during nanoparticle synthesis likely has contributed to the observance of the signal peak in FeNPs. As shown in Table 3, the biosynthesized FeNPs in DI solvent contain elements including C, O, and Fe with their corresponding percentage weights as 50.78, 41.61, and 3.02 % wt, respectively. The composition of elements present in FeNPs biosynthesized in NMP solvent includes C, O, and Fe contents with 41.49, 37.75, and 11.53 wt %, respectively, as listed in Table 4. This decrease in organic content in NMP might be due to more interaction of organic residues promoting FeNPs formation. It is noteworthy that the emergence of elements Cu, K, and P in the spectrum may stem from trace contributions from various sources. The appearance of trace Cu signal most likely has raised from instrumental background or cross-contamination from prior samples such as from the copper grid or sample holder used during analysis. Notably, the low-intensity signal of the Cu peak suggests that it does not significantly influence the overall nanoparticle composition or interfere with the interpretation of FeNP formation. Furthermore, the presence of K and P can be reasonably attributed to the use of green tea extract as a reducing agent, especially those rich in natural phytochemicals and polyphenols often contain trace amounts of mineral elements including K and P.

**Figure 7:**
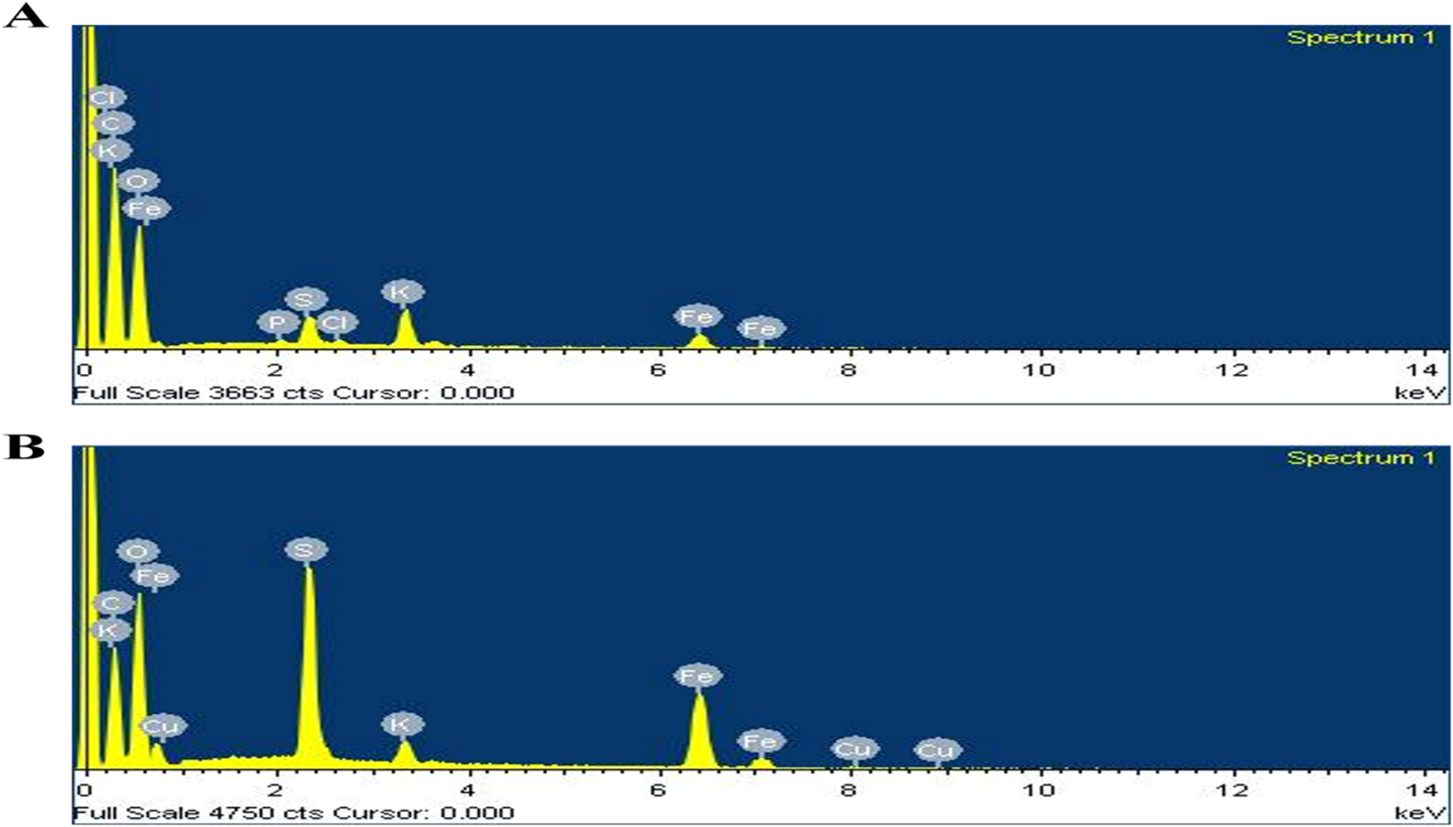
EDX spectra of biosynthesized FeNPs in (A) DI and (B) NMP solvents.

**Table 3:** Elemental Composition of FeNPs in DI

element	weight %	atomic %
C	50.78	60.30
O	41.61	37.09
P	0.23	0.10
S	1.55	0.69
Cl	0.31	0.12
K	2.51	0.92
Fe	3.02	0.77
total	100	

**Table 4:** Elemental Composition of FeNPs in NMP

element	weight %	atomic %
C	41.49	54.86
O	37.75	37.47
S	7.77	3.85
K	1.13	0.46
Fe	11.53	3.28
Cu	0.34	0.08
Total	100	

Zeta size analysis was carried out to determine the hydrodynamic size of the biosynthesized FeNPs. The variations in the intensity of light dispersed from nanoparticle solutions are utilized to calculate the average particle size. In Figure 8A, the average particle size distribution of biosynthesized FeNPs in DI solvent was observed to be 78.31 d.nm with a polydispersity index of 0.625. This size distribution is due to the adsorption of molecular H_2_O from the solvent.^46^ Additionally, in NMP, the average particle size was calculated to be 85 d.nm with a polydispersity index of 0.702 as shown in Figure 8B. The larger size scale of the nanoparticles with a higher polydispersity index suggested that FeNPs were formed in varying sizes. This was confirmed by the emergence of a less intense size distribution peak as shown in Figure 8B. Aggregation of nanoparticles observed in the samples also confirmed the relatively broad size distribution.^47^

**Figure 8:**
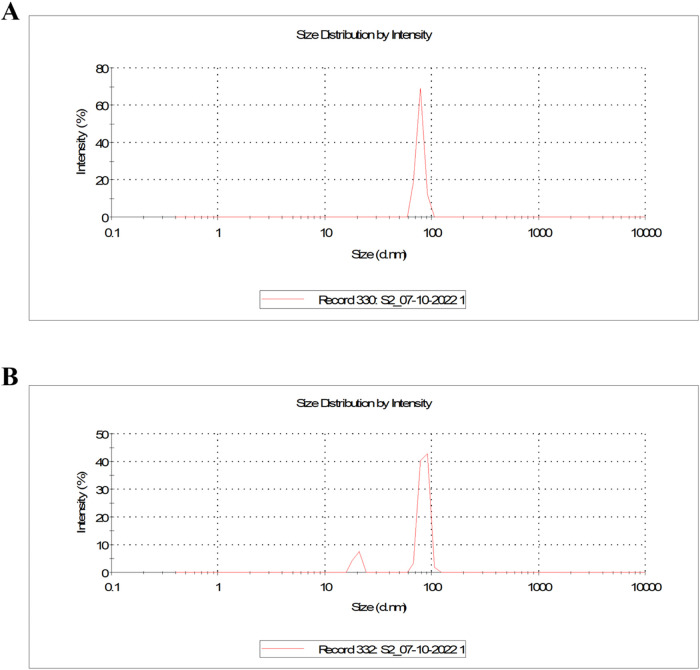
Measurements from zeta sizer showing the size of biosynthesized FeNPs in (A) DI solvent and (B) NMP solvent.

As illustrated in Figure 9, the FTIR spectrum of the biosynthesized FeNPs using green tea extract reveals a diverse array of functional groups that significantly influence their physicochemical properties. The FTIR spectra of green tea extract, as depicted in Figure 9, identified a broad adsorption band at 3412 cm^–1^ corresponding to the O–H stretching. Additionally, the transmittance dips found at 2922 and 2851 cm^–1^ correspond to the vibrational stretching of the C–H group in alkanes and the O–H stretch in carboxylic groups present in the extract, respectively. A discrete peak was observed at 1636 cm^–1^ which was associated with C=O stretch in polyphenols and C=C stretching in the aromatic compounds. Distinct absorption features at fingerprint regions of 1453, 1374, and 1239 cm^–1^ were indicative of the presence of methylene C–H bend, carboxylic groups, and amide III band, respectively. Furthermore, the transmittance bands at 1146 and 1101 cm^–1^ correspond to the C–O stretch of secondary alcohols. The minor dips at the lower frequency region of 874 cm^–1^ are assigned to H out-of-plane bending alongside peaks at 761, 609, and 558 cm^–1^ which are due to C=Cl, C=Br, and C=I, respectively, suggesting the presence of aliphatic compounds. A comparative analysis of transmittance spectra of FeNPs with the green tea extract revealed peaks at 3398, 2925, and 2852 cm^–1^, indicating the presence of O–H stretch, C–H stretch in alkanes, and O*–*H stretch by carboxylic groups, respectively. Additionally, the shift in peak from 1636 cm^–1^ to a sharp peak at 1628 cm^–1^ is exhibited in the FeNPs spectra, which indicates that the polyphenols and aromatic compounds attributed to this band were likely responsible for both the reduction and the surface functionalization of FeNPs. Furthermore, the dip observed at 1115 cm^–1^ corresponding to the C–O stretch of secondary alcohol affirmed that these functional groups are closely aligned with those observed in the green tea extract, confirming that the nanoparticle surface is coated with active biomolecules from green tea extract.^48,49^

**Figure 9:**
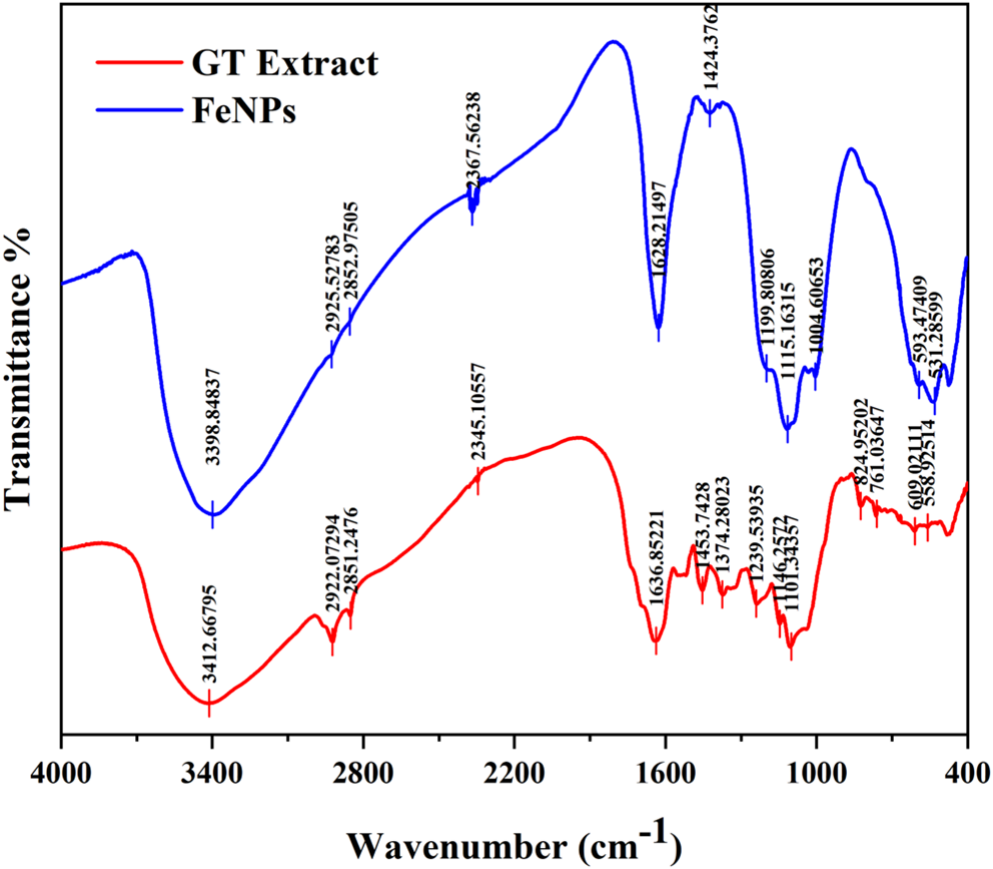
FTIR spectra of green tea extract and biosynthesized FeNPs.

Although *N*-methyl-2-pyrrolidone (NMP) is an effective solvent for nanoparticle synthesis, it poses significant environmental and toxicity risks, necessitating strict regulatory oversight and mitigation strategies. Environmentally, NMP is biodegradable under aerobic conditions, but degradation rates may vary depending on water systems with faster degradation in river water relative to wetlands or springs, leading to prolonged environmental exposure in slower-moving water systems.^50^ However, industrial discharges can present risks to contaminate surface water, groundwater, and soil.^51^ Classified as a reproductive toxicant (Category 1B), it can cause fetal developmental delays, as found in animal studies. In humans, NMP is not classified as carcinogenic but long exposure reported irritation and headache in workplace studies.^52^ It is also highly prone to sonochemical degradation during processing, generating byproducts that are difficult to characterize and may contaminate the nanomaterials. This degradation alters NMP’s optical properties, increasing scattering and photoluminescence, which can interfere with spectroscopic measurements. Additionally, the yield and exfoliation efficiency in NMP depend on the dissolved oxygen and water content, making the process less predictable. Degradation during exfoliation can further affect NMP’s performance, leading to inconsistencies in surface tension and solubility measurements. These challenges complicate solvent selection based on solubility parameters and contribute to batch-to-batch variations in nanoparticle properties.^53^

## 4 Conclusions

This study reports the role of solvents: DI and NMP in a facile, eco-friendly, green-mediated synthesis of FeNPs. A comprehensive suite of characterization techniques, including UV–visible spectroscopy, XRD, FESEM, and FTIR, was employed to systematically analyze the optical behavior, structural configuration, and morphological features of the synthesized nanoparticles. The spectral features in the UV region point to ligand-to-metal charge transfer processes, involving excitation from O 2p to Fe 3d orbitals while the broad visible band further supports the presence of double exciton processes, likely driven by strong Fe^3+^–Fe^3+^ electronic interactions. The oxidation of NMP to 5-hydroxy-*N*-methyl-2-pyrrolidone via peroxide intermediates subsequently promotes nanoparticle formation by reducing metal ions. An increase in band gap values from the bulk revealed that the synthesized nanoparticles showed potential for optoelectronic behavior. Additionally, mixed phases of α-Fe_2_O_3_ and γ-Fe_2_O_3_ structures are obtained from crystallographic analysis. At ambient room temperature, nucleation and growth of nanoparticles were favorable, revealing mean particle sizes of 52.20 ± 14.65 and 51.77 ± 13.82 nm in DI and NMP, respectively. Furthermore, FTIR analysis confirmed the presence of active functional groups from green tea extract, likely responsible for reducing and functionalization of FeNPs. Overall, this study brought forward novel insights into the effects of solvent on understanding nanoparticle formation mechanisms and further clarified the role of solvent interaction in FeNPs.

## Data Availability

All of the data set related to the research work performed have already been added to the manuscript.
